# Ocean acidification alters morphology of all otolith types in Clark’s anemonefish (*Amphiprion clarkii*)

**DOI:** 10.7717/peerj.6152

**Published:** 2019-01-07

**Authors:** Robert J. Holmberg, Eric Wilcox-Freeburg, Andrew L. Rhyne, Michael F. Tlusty, Alan Stebbins, Steven W. Nye Jr., Aaron Honig, Amy E. Johnston, Christine M. San Antonio, Bradford Bourque, Robyn E. Hannigan

**Affiliations:** 1 School for the Environment, University of Massachusetts Boston, Boston, MA, United States of America; 2 Department of Biology, Marine Biology and Environmental Science, Roger Williams University, Bristol, RI, United States of America; 3 Center for Economic and Environmental Development, Roger Williams University, Bristol, RI, United States of America

**Keywords:** Ocean acidification, Fish otoliths, CaCO_3_ mineralogy, Scanning Electron Microscopy

## Abstract

Ocean acidification, the ongoing decline of surface ocean pH and [CO${}_{3}^{2-}$] due to absorption of surplus atmospheric CO_2_, has far-reaching consequences for marine biota, especially calcifiers. Among these are teleost fishes, which internally calcify otoliths, critical elements of the inner ear and vestibular system. There is evidence in the literature that ocean acidification increases otolith size and alters shape, perhaps impacting otic mechanics and thus sensory perception. Here, larval Clark’s anemonefish, *Amphiprion clarkii* (Bennett, 1830), were reared in various seawater pCO_2_/pH treatments analogous to future ocean scenarios. At the onset of metamorphosis, all otoliths were removed from each individual fish and analyzed for treatment effects on morphometrics including area, perimeter, and circularity; scanning electron microscopy was used to screen for evidence of treatment effects on lateral development, surface roughness, and vaterite replacement. The results corroborate those of other experiments with other taxa that observed otolith growth with elevated pCO_2_, and provide evidence that lateral development and surface roughness increased as well. Both sagittae exhibited increasing area, perimeter, lateral development, and roughness; left lapilli exhibited increasing area and perimeter while right lapilli exhibited increasing lateral development and roughness; and left asterisci exhibited increasing perimeter, roughness, and ellipticity with increasing pCO_2_. Right lapilli and left asterisci were only impacted by the most extreme pCO_2_ treatment, suggesting they are resilient to any conditions short of aragonite undersaturation, while all other impacted otoliths responded to lower concentrations. Finally, fish settlement competency at 10 dph was dramatically reduced, and fish standard length marginally reduced with increasing pCO_2_. Increasing abnormality and asymmetry of otoliths may impact inner ear function by altering otolith-maculae interactions.

## Introduction

Since the advent of the industrial revolution, humankind has inadvertently relocated a significant volume of carbon to the troposphere, where it now resides as a greenhouse gas, warming the earth via radiative forcing (IPCC, 2013). Global warming, however, is not the sole consequence of surplus atmospheric CO_2_: the surface ocean has absorbed approximately 30% of anthropogenic CO_2_ emissions (Mikaloff Fletcher et al., 2006; Le Quéré et al., 2010), contributing to ocean acidification (Caldeira & Wickett, 2003). While this absorption is an important sink, abating the greenhouse effect (IPCC, 2013), it has consequences for marine ecosystems. Following diffusion, aqueous CO_2_ impacts seawater chemistry by reducing pH and carbonate (CO${}_{3}^{2-}$) concentration (Doney et al., 2009). Both will impact the fitness of marine biota, with cascading effects up to the ecosystem level (Fabry et al., 2008; Queirós et al., 2015; Hoegh-Guldberg et al., 2017). From population abundances to community shifts, ocean acidification has the potential to alter the ecological landscape of the ocean (Gaylord et al., 2015).

The declining availability of free CO${}_{3}^{2-}$ is particularly worrisome due to its implications for marine calcifiers, which use calcium carbonate (CaCO_3_) to form body structures including shells, teeth, and spines. Surface waters are normally supersaturated with CO${}_{3}^{2-}$, but as [CO${}_{3}^{2-}$] decreases, calcifiers may struggle to precipitate CaCO_3_ (Gattuso & Buddemeier, 2000). Furthermore, if seawater is undersaturated with respect to calcium carbonate minerals (e.g., aragonite, Ω_Ar_), existing structures may readily dissolve (Orr et al., 2005). A vast body of literature expounds ocean acidification’s anticipated effects on calcifier fitness in the future ocean, demonstrating variable degrees of severity (Hendriks, Duarte & Álvarez, 2010; Kroeker et al., 2013). Differential responses may depend on the specific biochemical pathways involved in calcification (Ries, Cohen & McCorkle, 2009), biological mechanisms for buffering pH changes in body fluids (Munday et al., 2011a), energetics limiting physiological acclimation (Seibel, Maas & Dierssen, 2012), or various ecological forces acting on an organism (Kroeker, Micheli & Gambi, 2012).

Teleostei is an extremely diverse infraclass of Actinopterygii representing the modern bony fishes, comprised of more than 30,000 species and dominating most aquatic habitats (Froese & Pauly, 2018). Teleosts are internal calcifiers, precipitating CaCO_3_ in the intestinal lumen that aids water absorption and osmoregulation (Grosell, 2011), and precipitating otoliths in the inner ear that are critical for mechanoreception (Moyle & Cech, 2004). Heuer & Grosell (2014) reviewed numerous effects of acidification on marine teleosts, including respiratory acidosis leading to sustained elevation of blood plasma HCO${}_{3}^{-}$ (Esbaugh, Heuer & Grosell, 2012), cognitive disruption and behavioral changes linked to inhibited GABA_A_ neurotransmitter receptor function (Nilsson et al., 2012), mixed impacts on standard and maximum metabolic rates with implications for aerobic scope (Munday, Crawley & Nilsson, 2009), and increased otolith area (Munday et al., 2011b) and mass (Bignami, Sponaugle & Cowen, 2013; Bignami et al., 2013). As such, otoliths may be points of vulnerability for teleosts in the near-future ocean (Ishimatsu, Hayashi & Kikkawa, 2008; Munday et al., 2008; Heuer & Grosell, 2014).

Otoliths, or ear stones, are critical features located within the inner ear of teleost fishes, formed by precipitation of CaCO_3_ around a protein-rich matrix and bathed in endolymph (Panella, 1971). CaCO_3_ supersaturation is maintained in the endolymph by proton pumps in the epithelial cells adjacent to the site of crystallization, which maintain the pH gradient required for CO${}_{3}^{2-}$ - HCO${}_{3}^{-}$ balance (Ishimatsu, Hayashi & Kikkawa, 2008). Otoliths exist in three pairs (sagittae, lapilli, asterisci), with one from each pair contained within each otolithic end organ. When disturbed by fish movement or sound waves, otoliths trigger sensory maculae lining the interior wall of their chambers, converting the force into electrical impulses interpreted by the brain. Likewise, otoliths function as sensory organs for hearing and gravisense (Popper & Fay, 1993).

Researchers recognize the potential for ocean acidification to impact otolith growth in teleosts, especially during the sensitive larval phase, and many have demonstrated effects experimentally (Table 1). Contrary to the hypothesis that ocean acidification will inhibit otolith growth due to dwindling CO${}_{3}^{2-}$ availability (Ishimatsu, Hayashi & Kikkawa, 2008), elevated seawater pCO_2_ stimulates growth of sagittae and/or lapilli in many taxa. This growth is attributed to elevated blood plasma [HCO${}_{3}^{-}$], retained to buffer acidosis and transported into the endolymph where it becomes substrate for CO${}_{3}^{2-}$ aggregation (Checkley et al., 2009; Munday et al., 2011b; Heuer & Grosell, 2014). Only one study (Mu et al., 2015) observed decreased otolith size in response to elevated pCO_2_. Other studies (Franke & Clemmesen, 2011; Munday et al., 2011a; Simpson et al., 2011; Frommel et al., 2013; Perry et al., 2015; Cattano et al., 2017; Martino et al., 2017; Jarrold & Munday, 2018) observed no effects of pCO_2_ on otolith morphology.

**Table 1:** Summary of observed ocean acidification impacts on otolith morphology. In the ‘Metrics’ column, S denotes effects of pCO_2_ on sagittae and L denotes effects on lapilli. Metrics that increased at elevated pCO_2_ are designated with an up arrow; metrics that decreased at elevated pCO_2_ are designated with a down arrow. The ‘Min. Effect’ column represents the minimum pCO_2_ threshold for which any effect was observed, reported to the decimal place published.

**Citation**	**Species**	**Life Stage**	**Metrics**	**Min. Effect (µatm)**
Checkley et al. (2009)	*Atractoscion nobilis*	Larval	↑ S Area	993
Munday et al. (2011b)	*Amphiprion percula*	Larval	↑ S Area, Length	1,721.4
Hurst et al. (2012)	*Theragra chalcogramma*	Juvenile	↑ S Mean Incr. Width	478
Bignami, Sponaugle & Cowen (2013), Bignami et al. (2013)	*Rachycentron canadum*	Larval	↑ S Mass; ↑ S,L Area, Vol., Dens., ↓ Area/Vol.	800
Maneja et al. (2013)	*Gadus morhua*	Larval	↑ S,L Area; ↑ S Roundness; ↓ L Roundness	1,800
Bignami, Sponaugle & Cowen (2014)	*Coryphaena hippurus*	Larval	↑ S,L Area	1,190
Pimentel et al. (2014)	*Solea senegalensis*	Larval	↑ S Area	1,600
Schade, Clemmesen & Wegner (2014)	*Gasterosteus aculeatus*	Juvenile	↑ S Area	1,167
Mu et al. (2015)	*Oryzias melastigma*	Larval	↓ S Area	2,372.6
Réveillac et al. (2015)	*Sparus aurata*	Juvenile	↑ S Calc. Rate, Area/TL, ↓ Roundness	726
Shen et al. (2016)	*Atractoscion nobilis*	Larval	↑ S,L Area	2,500
Faria et al. (2017)	*Argyrosomus regius*	Larval	↑ S Area, Perimeter, Width	1,900
	*Diplodus sargus*	Larval	↑ S Area, Perimeter	1,100
	*Solea senegalensis*	Larval	↑ S Area, Perimeter	1,900
Martins (2017)	*Lepadogaster lepadogaster*	Larval	↑ S Roundness	1,541.68
Mirasole et al. (2017)	*Diplodus vulgaris*	Juvenile^a^	↑ S Relative Length; Altered Shape	pH 7.8^b^
	*Gobius bucchichi*	Adult^a^	S Altered Shape	pH 7.8^b^
Coll-Lladó et al. (2018)	*Sparus aurata*	Larval	↑ S,L Area, Perimeter, Shape Irregularity	1,159

**Notes.**

a Life stage, although unlisted in the manuscript, is here inferred from fish standard length (SL).

b pCO_2_ is unlisted in the manuscript and cannot be calculated without additional seawater carbonate chemistry parameter(s).

Evidence that acidification alters otolith size and shape has inspired hypotheses that this could interfere with otic mechanics, and thus impair sensory perception in teleosts (e.g., Munday et al., 2011b; Bignami et al., 2013; Bignami, Sponaugle & Cowen, 2014). Indeed, there is some evidence that asymmetry of otolith size, shape, and mass may impair auditory/vestibular function in some species with consequences for habitat detection and overall fitness (Lychakov & Rebane, 2005; Gagliano et al., 2008; Anken, Knie & Hilbig, 2017). Others have added that increased otolith size from ocean acidification could enhance auditory sensitivity to the benefit or detriment of the fish depending on life history (Bignami et al., 2013; Bignami, Sponaugle & Cowen, 2014; Réveillac et al., 2015).

While most available studies quantified simple morphometrics to analyze pCO_2_ effects on otolith morphology, the most informative among them augmented morphometrics with other analyses, including complex shape analyses (e.g., Fourier analysis) (Munday et al., 2011a; Munday et al., 2011b; Simpson et al., 2011; Martino et al., 2017; Mirasole et al., 2017); mass, volume and density analyses (Bignami, Sponaugle & Cowen, 2013; Bignami et al., 2013); and compositional analyses (e.g., LA-ICPMS) (Munday et al., 2011b; Hurst et al., 2012; Martino et al., 2017; Mirasole et al., 2017; Coll-Lladó et al., 2018). Similarly, scanning electron microscopy can be used to screen for treatment effects on aspects of otolith morphology and composition that, although typically overlooked in simple morphometric analysis, may impact ear function. These may include: (i) lateral development, defined as the degree of convexity of an otolith’s lateral face; (ii) percent visible crystals, defined as an estimate of surface crystal density or grain, approximating surface roughness; (iii) crystal habit, here defined as any deviation in crystal shape from the predominant orthorhombic aragonite in sagittae and lapilli, or hexagonal vaterite in asterisci; and (iv) overall mineralogy, here defined as relative proportion of orthorhombic aragonite versus hexagonal vaterite visible on an otolith’s surface. The former two metrics estimate an otolith’s surface topography and texture, and the latter two estimate crystal features indicative of composition, density, and stability under environmental stress (Boulos et al., 2015). These metrics are intended as first-pass screening tools for efficiently identifying general trends in the data; should they yield compelling evidence of treatment differences, they could be followed with more rigorous methods to best quantify the variable (e.g., measuring otolith height directly or determining CaCO_3_ polymorph composition with Raman spectroscopy (Coll-Lladó et al., 2018)).

In addition to standard morphometrics, the mineralogical metrics described above were used to investigate ocean acidification impacts on otolith morphology in larval Clark’s anemonefish, *Amphiprion clarkii* (Bennett, 1830). *A. clarkii* is a teleost reef fish belonging to Pomacentridae and inhabiting shallow reefs throughout the Indo-Pacific (Froese & Pauly, 2018). The species was chosen both as a novel taxon and to enable intragenus comparison with previous work (Munday et al., 2011b). Any impacts on its otolith morphology could have implications for teleost sensory perception and fitness in the future ocean.

## Materials & Methods

### Livestock

All husbandry was completed at Roger Williams University in Bristol, Rhode Island, USA (IACUC #R-11-09-13). Several *Amphiprion clarkii* (Bennett, 1830) broodstock pairs were reared, all wild-caught in Fiji and acquired from Long Island Aquarium, Riverhead, New York, USA. Broodstock periodically laid clutches of eggs on porcelain tiles in aquaria (every 10–12 days). One large, healthy clutch was selected from a single broodstock pair, removed the night before anticipated day of hatch (around day eight post-deposition), and placed in a separate, aerated, 200 L hatching aquarium. Upon hatch, *A. clarkii* larvae were randomly distributed into 40 L experimental aquaria at a density of 40 individuals per aquarium. Throughout the experimental trial, larvae were fed ad libitum with wild copepods from monoculture (*Pseudodiaptomus spp.*) in a background of algae (*Isochrysis spp.*). *Pseudodiaptomus spp.* were dosed to densities of 5 mL^−1^ and 1 mL^−1^ (nauplii and adults respectively), as measured using a counting wheel, and *Isochrysis spp.* twice daily to maintain a concentration of 40,000 cells mL^−1^, as measured using a cell counter (Beckman Coulter Inc., Brea, CA).

### Experimental trial

The experimental design consisted of four pCO_2_/pH treatments selected to model various present and anticipated future ocean conditions: (i) 350 µatm/pH 8.16 (control), modern ocean conditions; (ii) 800 µatm/pH 7.80, approximate conditions projected for 2100 under Representative Concentration Pathway (RCP) 8.5 (IPCC, 2013); (iii) 1,600 µatm/pH 7.60, nearly double 2,100 levels under RCP 8.5 (IPCC, 2013); (iv) 3,000 µatm/pH 7.30, a reasonable extreme given coastal eutrophication-induced acidification (Wallace et al., 2014), and given that eutrophication is already occurring in some reef systems inhabited by *A. clarkii* (Bell, Elmetri & Lapointe, 2014; Fabricius, 2005). Treatments were replicated three times and assigned to 12 experimental units (aquaria) in a randomized design. Seawater was sourced from Mt. Hope Bay, sterilized using sodium hypochlorite and UV light, filtered to 1 µm, and used to fill experimental aquaria. 25% water changes were completed every other day using drip buckets at 100 mL min^−1^. Seawater salinity and temperature were measured twice daily in all aquaria using a handheld meter (YSI, Yellow Springs, OH). Seawater total alkalinity was measured once every other day in all aquaria using a tabletop autotitrator (Hanna Instruments, Smithfield, RI). The experimental trial took place within an environmental chamber to maintain ambient air conditions at 28 °C, and aquaria were covered with loose fitting lids to minimize CO_2_ outgassing and evaporative heat loss. Seawater was aerated with house-supplied air connected to airstones to maintain dissolved oxygen. Experimental treatments were achieved and maintained by dosing CO_2_ gas through the airstones using a CO_2_ dosing apparatus (Wilcox-Freeburg et al., 2013) controlled by hobbyist aquarium controllers (Digital Aquatics, Woodinville, WA). pH_T_ of each aquarium was measured continuously using research-grade glass combination electrodes calibrated to synthetic seawater buffers (Byrne, 1987; Millero et al., 1993), prepared from analytical reagent grade chemicals (Fisher Scientific, Hampton, NH). The aquarium controller output pH_T_ data every 1-3 s via RSS feed, which was parsed/logged to a PC with custom Perl and MATLAB scripts (Wilcox-Freeburg, 2014) (Perl Version 5.28.0, https://www.perl.org/; MATLAB Version R2017b, https://www.mathworks.com/products/matlab.html). Average DIC, pCO_2_, and Ω_Ar_ were calculated for each aquarium from measured seawater parameters using CO2calc (https://soundwaves.usgs.gov/2011/03/research4.html). The experimental trial concluded after 10 days, at the onset of fish metamorphosis.

### Data collection

Upon conclusion of the experimental trial, and following euthanization of fish with a lethal dose of tricaine mesylate (MS-222) in seawater, each individual was counted, placed on a Sedgewick rafter (1 mm), and photographed with a digital camera-equipped stereomicroscope at 10×–90× magnification. Mortality counts (by aquarium) were calculated by subtracting final fish counts from initial stocking density. Standard lengths of each individual were measured to 1/100 mm from stereomicrographs with ImageJ (Version 1.51n; https://imagej.nih.gov/ij/), and averaged by aquarium (arithmetic mean). Settlement competency was determined according to behavioral and morphological criteria; larvae were considered competent to settle when they began to cling to an aquarium wall rather than swim freely in the water column, and concurrently develop pigmentation consistent with settlement-stage metamorphosis. Due to natural variance in ontogeny among individuals, some had not achieved settlement competency when the experimental trial ended at 10 days post hatch (dph). Proportions of surviving fish that achieved settlement competency by 10 dph versus those that had not were tallied for each aquarium. Non-settlement-stage fish data (standard length, otolith morphometric variables, otolith mineralogical variables) were excluded from all further analyses (sample exclusion criteria were pre-established; 38 fish, or roughly 15% of surviving fish, were excluded). Next, all six otoliths (two each of sagittae, lapilli, and asterisci) were manually removed under a polarizing stereo dissecting microscope. Each set of otoliths was digitally photographed with the stereomicroscope at 90x magnification and mounted to aluminum scanning electron microscopy (SEM) stubs for later analysis. Area, perimeter, major axis, and minor axis of all six otoliths from all fish were quantified from stereomicrographs with custom MATLAB image analysis software (Wilcox-Freeburg, 2014) (MATLAB Version R2017b; https://www.mathworks.com/products/matlab.html). All otolith morphometrics were measured to 1/100 unit. Otolith circularity was calculated from major and minor axes ($ \frac{\pi \times (\text{minor axis}/2)^{2}}{\pi \times (\text{major axis}/2)^{2}} $). Aquarium means for each morphometric variable were determined by calculating the arithmetic mean of data from all individual fish within each aquarium (grouped by otolith type and side). Due to moderate-strong correlations between standard length and otolith area and perimeter at the evaluation unit (individual fish) level (area: *r* > 0.49, perimeter: *r* > 0.30 for all otolith types/sides), otolith area and perimeter were normalized to standard length of individuals prior to calculating aquarium means. This facilitated investigation of treatment effects while accounting for potentially confounding differences in standard length between fish. Next, each individual otolith was imaged with SEM in secondary electron mode using a working distance of 10 mm, spot size of 30, accelerating voltage of 10 kV, and magnification up to 3,000×. Scanning electron micrographs were scored visually for various mineralogy-related variables multiple times using Qualtrics survey software (Version N/A; https://www.qualtrics.com/). Six trained, independent readers scored variables including lateral development (scale of 1–5), crystal habit (orthorhombic, hexagonal, acicular, acrystalline, amorphous), percent visible crystals (5–50%), and mineralogy (proportion aragonite/vaterite on an otolith’s surface interpretable by crystal habit) according to a rubric (see Supplemental Information 2 for the rubric used to train and guide readers through scoring^1^). 1“Core development” in the rubric has been renamed “lateral development” in the manuscript. In the rubric, “core” refers not to the otolith’s core but to the center of its lateral face.The rubric contains reference illustrations (and in the case of the lateral development variable, micrographs) for each metric and lists categories to choose from for scoring; for each metric, the readers were asked to choose the option that best categorizes each otolith. Poor-quality micrographs due to mounting errors or broken otoliths were marked as unusable and not scored (41 micrographs, or roughly 4% of all micrographs, were excluded). Otolith-specific raw data grouped by type and side were generated for each variable from the survey questions. Each individual otolith was assigned the mode of survey scores for each variable. If a two-way mode tie occurred, the lower of the modes was selected. If a three-way mode tie occurred, the median mode was selected. For the lateral development and percent visible crystals variables, aquarium means were determined by calculating the arithmetic mean of the survey response mode for each otolith type and side, thus generating approximately continuous aquarium means from ordinal data (Norman, 2010). The mineralogy and crystal habit variables are nominal, so aquarium means were determined by calculating the mode of the otolith modes. See Data S1 for data means to be subset by otolith type/side and Data S2 for raw, fish-level data.

### Statistical analyses

Hypothesis tests were used to identify and interpret any impacts of increasing seawater pCO_2_ on otolith morphology in each otolith type/side, as well as on fish mortality, settlement competency, and standard length. All statistical analyses were conducted using R (Version 3.4.3; R Core Team, 2017; see Supplemental information 1 for analyses). Regression analyses (pCO_2_ as a continuous variable) were selected over ANOVA with multiple comparisons (pCO_2_ as a grouping factor) for their greater power, informativeness, parsimony, and appropriateness for interpreting linear dose–response relationships (Lazic, 2008). Polynomial models were considered for all regression analyses, and model selection performed using goodness of fit tests (i.e., *F*-tests for general linear models and chi-squared tests for generalized linear models). All binomial logistic regression models were tested for overdispersion. All statistical tests were two-tailed. The crystal habit and mineralogy response variables exhibited no variance across any treatment and otolith type, so they were excluded from further analyses. Principal component analysis (PCA) was performed on aquarium means for each otolith type and side. PCA was run on the correlation matrix between the morphometric (area, perimeter, circularity) and survey (lateral development, percent visible crystals) response variables using varimax rotation. Components with eigenvalues greater than or equal to 1.0 were retained. Treatment effects of pCO_2_ on otolith morphometrics were investigated with regression analysis, with component scores as the response variables and pCO_2_ as the explanatory variable. Regression analysis was performed on all components representing all otolith types and sides, and models in which pCO_2_ predicted component scores (*p* < 0.05) were retained. Treatment effect on fish mortality was investigated with binomial logistic regression analysis (link function = logit), with the proportion of mortality counts (per aquarium)/aquarium stocking density as the response variable and pCO_2_ as the explanatory variable. Treatment effect on somatic growth was investigated with regression analysis, with mean fish standard length (mm) as the response variable and pCO_2_ as the explanatory variable. Treatment effect on settlement competency at 10 dph was investigated with binomial logistic regression analysis, with the proportion of competent (per aquarium)/remaining fish (per aquarium) at the end of the experimental trial as the response variable and pCO_2_ as the explanatory variable. For all regression analyses that predicted an effect of pCO_2_, truncated models were created that are identical except that data from the 3,000 µatm pCO_2_/pH 7.30 treatment were excluded; this enabled interpretation of the models without the influence of this most extreme treatment, with the caveat that they employ n fewer degrees of freedom and are thus less powerful than the full models. For the truncated models, only *F*- and *p*-values are reported, as the full models were considered to be otherwise more informative.

## Results

### Seawater carbonate chemistry

pH treatments remained on target throughout the experimental trial (standard deviation ≤ 0.04) (Table 2).

**Table 2:** Seawater carbonate chemistry parameters. Values represent aquaria means (*n* = 3 for each treatment); standard deviations listed in parentheses for measured parameters.

Treatment (pH_T_)	S (ppt)	T (°C)	A_T_ (µmol kg^−1^)	DIC (µmol kg^−1^)	pCO_2_ (µatm)	Ω_Ar_
8.16 (0.04)	35.00 (0.30)	28.20 (0.40)	2,440 (147)	2,018	299.4	4.84
7.80 (0.01)	35.00 (0.30)	28.20 (0.40)	2,440 (152)	2,237	825.5	2.54
7.60 (0.01)	35.00 (0.30)	28.30 (0.40)	2,432 (140)	2,318	1,384.3	1.70
7.30 (0.01)	35.00 (0.30)	28.20 (0.40)	2,418 (140)	2,415	2,897.0	0.89

### Otolith morphometrics and scoring

**Figure 1:**
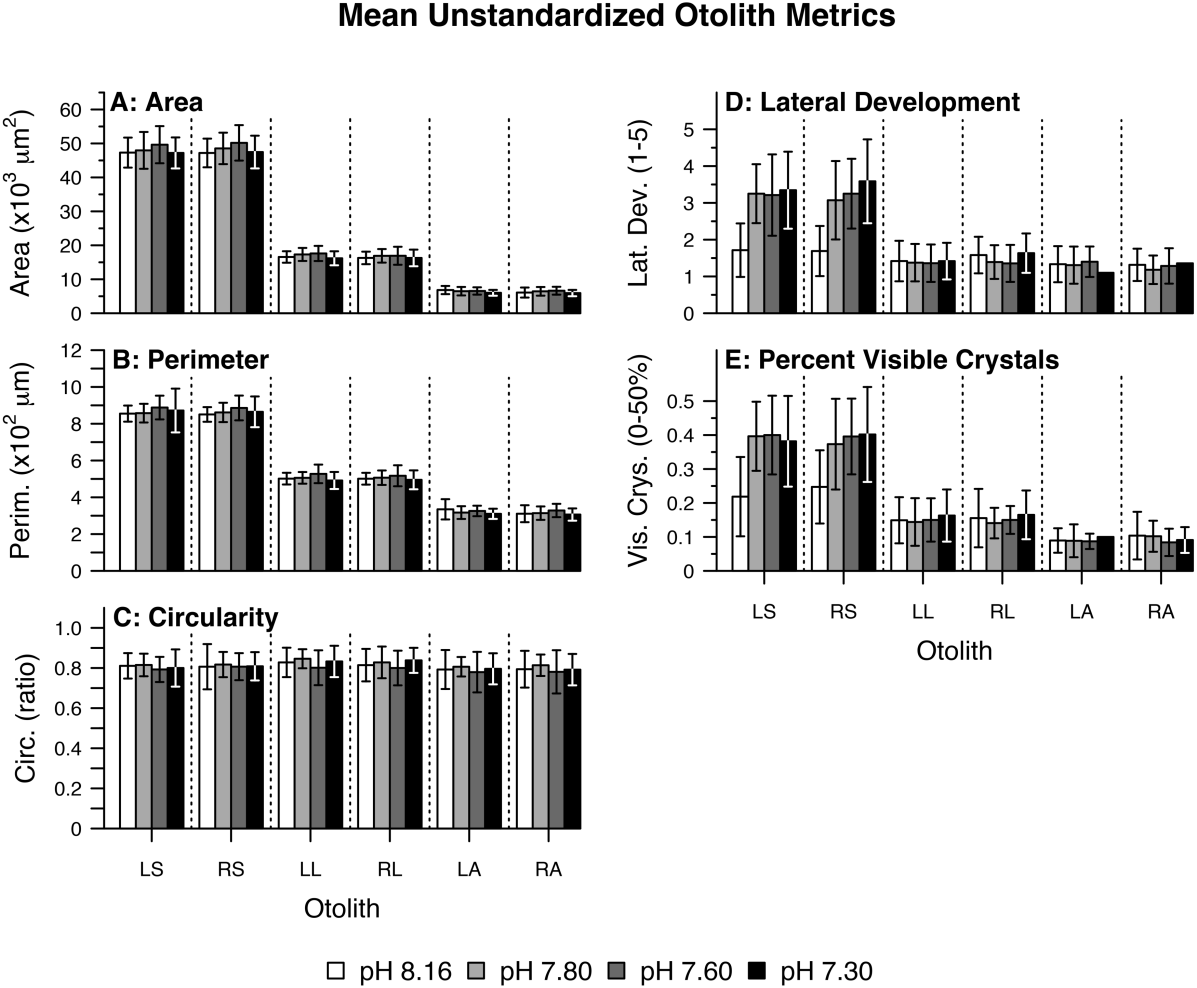
Mean unstandardized otolith metrics. Summary of raw (fish-level) otolith morphological data including (A) Area, (B) Perimeter, (C) Circularity, (D) Lateral Development, and (E) Percent Visible Crystals by pH/pCO_2_ treatment (legend) and otolith type/side. Otolith name abbreviations are identical to those elsewhere in the manuscript (e.g., LS for Left Sagittae). Bars represent combined means. Lines represent one (pooled) standard deviation.

Otolith morphometrics and mineralogical metrics varied with pCO_2_ treatment and according to otolith type and side (Fig. 1). Scoring for the crystal habit and mineralogy metrics never deviated from the norm for any otolith type in any treatment (i.e., sagittae and lapilli were consistently scored as predominantly aragonitic, exhibiting orthorhombic crystal habit; asterisci were assumed to be vateritic despite exhibiting little to no identifiable crystal habit).

### Principal component analysis

**Figure 2:**
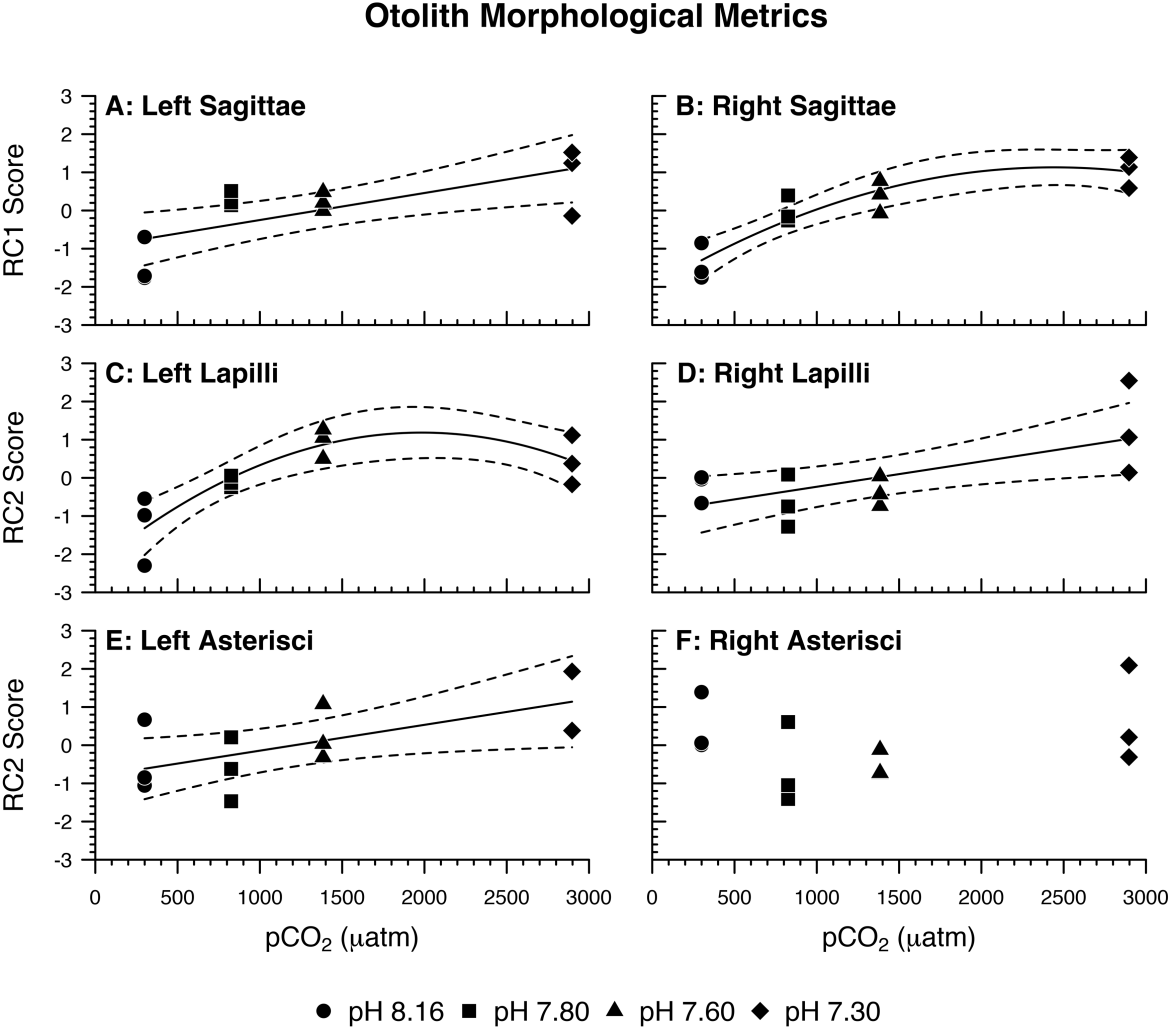
Otolith morphological metrics. Regression lines (solid) and 95% confidence bands (dotted) represent significant relations between pH/pCO_2_ treatment (legend) and (A, B, C, D, E) rotated component (RC) scores representing *Amphiprion clarkii* otolith morphological variables, grouped by otolith type and side (A, *p* = 0.0061; B, *p* = 0.0004; C, *p* = 0.0045; D, *p* = 0.0168; E, *p* = 0.0420). Right asterisci components vs. pCO_2_ did not yield significant relations, but RC2 scores are plotted for illustrative consistency. Data points represent aquaria. *N* = 12, *n* = 3 except where (E) no data is available for an aquarium (*N* = 11, *n* = 2 for pH 7.30 treatment only). See Table 3 for otolith morphological variables and corresponding PCA loadings.

For each otolith type and side, principal component analysis produced two components with eigenvalues greater than 1.0 (Table 3). From here on, the components are referred to as rotated components—RC1 or RC2—to reflect that their loadings were scaled with varimax rotation. *Left Sagittae (LS)*: As pCO_2_ increased, left sagittae were rendered overall larger and wider in circumference, with more pronounced lateral faces and rougher surface textures owed to greater visible crystal density (Tables 3 and 4; Fig. 2A). *Right Sagittae (RS)*: As pCO_2_ increased, right sagittae responded according to the same metrics as left sagittae, albeit with slightly stronger responses of area/SL and perimeter/SL (Tables 3 and 4; Fig. 2B). Whereas left sagittae responses were best represented as a linear model, right sagittae responses were best represented as a curvilinear (quadratic) model, with responses leveling out between the 1,600 µatm pCO_2_/pH 7.60 and 3,000 µatm pCO_2_/pH 7.30 treatments. *Left lapilli (LL)*: As pCO_2_ increased, left lapilli were rendered larger and wider in circumference (Tables 3 and 4; Fig. 2C). *Right Lapilli (RL)*: In the most extreme pCO_2_ treatment only, right lapilli were rendered rougher with more pronounced lateral faces despite remaining approximately the same size (Tables 3 and 4; Fig. 2D). Whereas left lapilli responses were best represented as a curvilinear (quadratic) model, with responses leveling out between the 1,600 µatm pCO_2_/pH 7.60 and 3,000 µatm pCO_2_/pH 7.30 treatments, right lapilli responses were best represented as a linear model. *Left Asterisci (LA)*: In the most extreme pCO_2_ treatment only, left asterisci were rendered increasingly elliptical (rather than circular), rougher, and wider in circumference (Tables 3 and 4; Fig. 2E). This was the only instance of 2-dimensional otolith shape change observed in response to treatment, as well as the only otolith metric that decreased rather than increased with increasing pCO_2_. *Right Asterisci (RA)*: Right asterisci were not observed to respond to increasing pCO_2_ (Fig. 2F). This was the only otolith type/side that exhibited no response to treatment.

**Table 3:** Component variances and loadings. Loadings corresponding to various Amphiprion *clarkii* otolith morphological parameters, the variance of which composes the rotated components in Fig. 2 and other components excluded from the analysis. Also included are the variances associated with each component and the total variance associated with components.

Otolith	Component	Variance (%)	Area/SL	Perimeter/SL	Circularity	Lateral development	Percent visible crystals
Left Sagittae	RC1^*^	47	0.62^*^	0.37	0.05	0.97^*^	0.94^*^
	RC2	37	0.61	0.85	−0.86	0.08	0.17
	Total	84					
Right Sagittae	RC1^*^	59	0.77^*^	0.72^*^	0.00	0.95^*^	0.96^*^
	RC2	29	0.39	0.61	−0.94	0.06	−0.07
	Total	88					
Left Lapilli	RC1	46	−0.12	−0.30	0.67	0.90	0.96
	RC2^*^	36	0.89^*^	0.90^*^	−0.32	−0.29	−0.03
	Total	82					
Right Lapilli	RC1	44	0.88	0.99	−0.65	−0.08	−0.01
	RC2^*^	34	0.02	0.06	0.31	0.88^*^	0.92^*^
	Total	78					
Left Asterisci	RC1	44	0.81	0.81	−0.15	−0.75	−0.54
	RC2^*^	34	−0.11	0.53^*^	−0.92^*^	0.04	0.73^*^
	Total	78					
Right Asterisci	RC1	50	0.89	0.98	−0.73	−0.06	0.48
	RC2	24	−0.16	0.08	−0.31	0.91	0.50
	Total	74					

**Notes.**

* signify which components are related to pCO_2_ (*p* < 0.05) and which variables are strongly associated with each of those components (*r* ≥ 0.50).

**Table 4:** Regression statistics. Regression models are listed by row, including all models in the manuscript for which pCO_2_ predicted the response variable (*p* ≤ 0.05), and truncated models with data from the 3,000 atm pCO_2_/pH 7.30 treatment excluded (designated by subscript T). For the otolith morphological models, model name abbreviations are identical to those elsewhere in the manuscript (e.g., LS for Left Sagittae). FSC stands for Fish Settlement Competency (at 10 dph). FSL stands for Fish Standard Length. Component names (Comp.) are listed for otolith morphological models only. Statistics including degrees of freedom (DF), *F*-statistics (*F*), *p*-values (*p*), line equations, line slopes multiplied by 100 (b_1_*100), 95% confidence intervals for slopes multiplied by 100 (CI*100), and *R*^2^s are listed. Only *DF*, *F*, and *p* are listed for truncated models, as the full models are considered to be more informative except for determining whether the 3,000 µatm pCO_2_/pH 7.30 treatment disproportionately weighted them. Line slopes and confidence intervals should be read as “[response variable] increased by [b_1_*100] [units] for every 100 µatm increase in pCO_2_ (95% CI: [CI*100])”. Line slopes and confidence intervals were excluded for polynomial (quadratic) models, as they are not as easily interpreted.

Model	Comp.	*DF*	*F*	*p*	Equation	b_1_*100	CI*100	*R*^2^
LS	RC1	1,10	11.98	0.0061	y = (7.28E−4)x − 0.98	0.07	0.03–0.12	0.50
LS_T_	RC1	1,7	10.67	0.0137	–	–	–	–
RS	RC1	2,9	20.56	0.0004	y = (2.51E−3)x − (5.08E−7)x^2^ − 1.98)	–	–	0.78
RS_T_	RC1	2,6	14.64	0.0049	–	–	–	–
LL	RC2	2,9	10.47	0.0045	y = (3.48E−3)x − (8.86*E* − 7)x^2^ − 2.25)	–	–	0.63
LL_T_	RC2	2,6	10.91	0.0100	–	–	–	–
RL	RC2	1,10	8.21	0.0168	y = (6.62E−4)x − 0.89	0.07	0.01–0.12	0.40
RL_T_	RC2	1,7	0.11	0.7489	–	–	–	–
LA	RC2	1,9	5.61	0.0420	y = (6.64E−4)x − 0.80	0.07	0.00–0.13	0.32
LA_T_	RC2	1,7	1.04	0.3423	–	–	–	–
FSC	–	1,10	4.83^a^	0.0279	logit (*π*) = (−3.81E−4)x + 2.31	0.04	0.00–0.07	0.38
FSC_T_	–	1,7	7.26^a^	0.0071	–	–	–	–
FSL	–	1,10	17.77	0.0018	y = (−1.31E−4)x + 6.85	0.01	0.00–0.02	0.60
FSL_T_	–	1,7	2.43	0.1629	–	–	–	–

**Notes.**

a For FSC and FSC_T_, which are binomial logistic regression models, the values listed as F are actually *χ*^2^-statistics.

### Mortality, settlement competency, and somatic growth:

**Figure 3:**
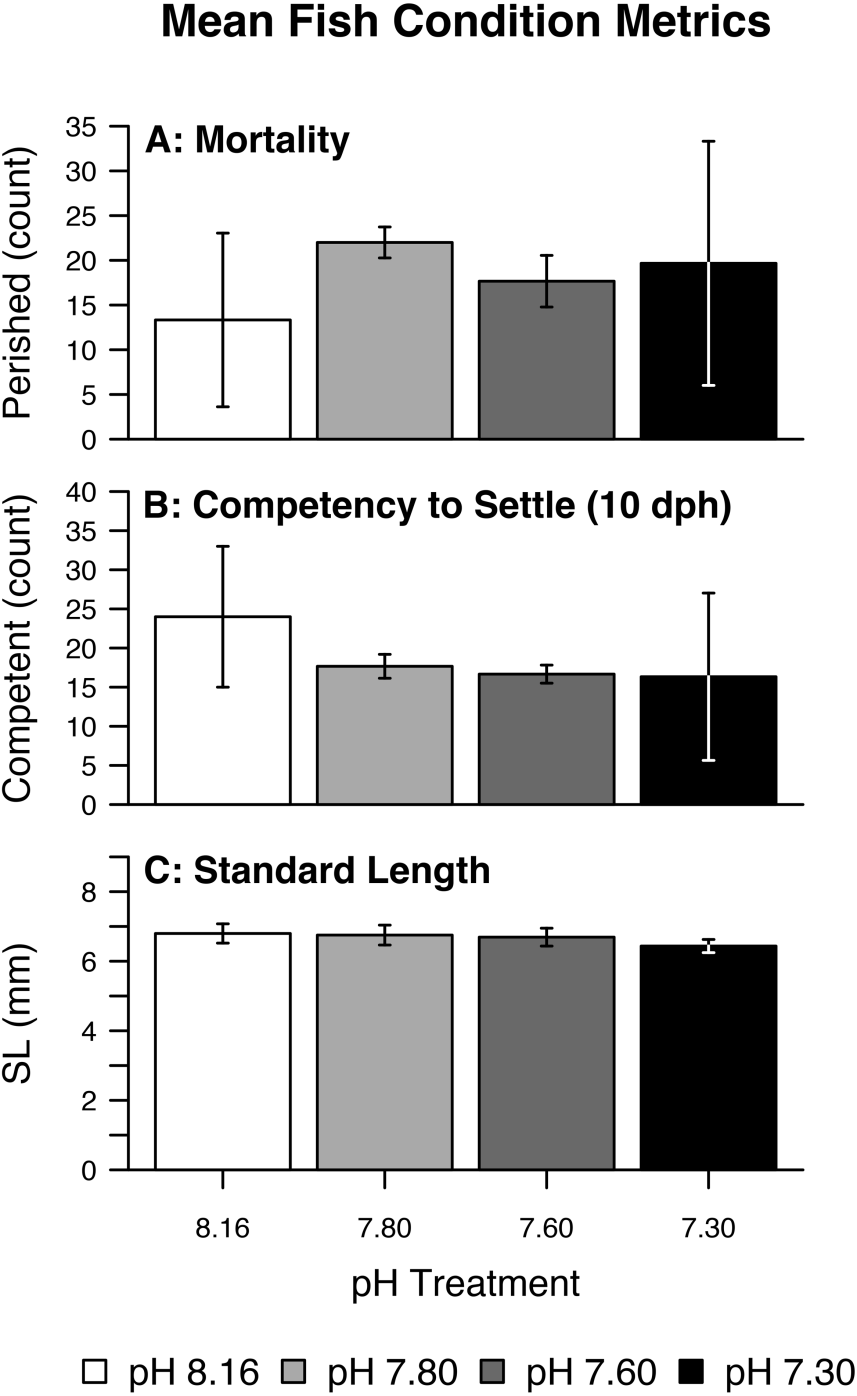
Mean fish condition metrics. Summary of raw (fish-level) fish condition data by pH/pCO_2_ treatment (legend) including (A) Mortality, (B) Competency to Settle at 10 days post-hatch (dph), (C) Standard Length. Bars represent combined means. Lines represent one (pooled) standard deviation.

**Figure 4:**
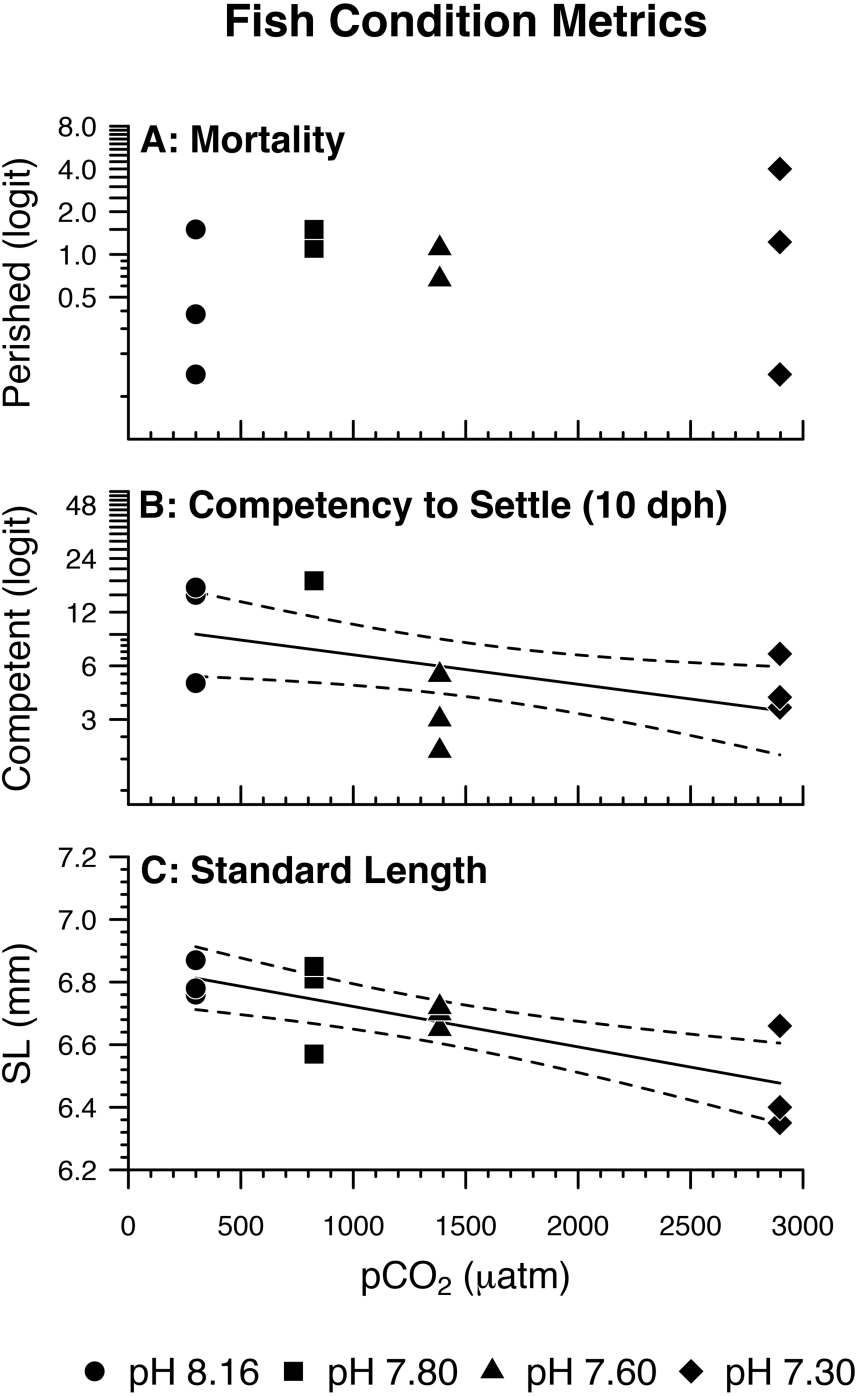
Fish condition metrics. (A) Odds of *Amphiprion clarkii* mortality by pH/pCO_2_ treatment (legend). Regression lines (solid) and 95% confidence bands (dotted) represent significant relations between pH/pCO_2_ treatment and (B) odds of on-time *A. clarkii* settlement (*p* = 0.0279); (C) *A. clarkii* standard length (*p* = 0.0018). Data points represent (A, B) binomial proportions by aquarium; (C) aquarium means. *N* = 12, *n* = 3 except where (B) 100% of fish in an aquarium settled on time (*N* = 10, *n* = 1 for pH 7.80 treatment only).

Fish mortality, competency to settle at 10 dph, and standard length varied somewhat with pCO_2_ treatment (Fig. 3). Despite high fish mortality throughout the experimental trial, mortality was not associated with pCO_2_ (Table 4, Fig. 4A). As pCO_2_ increased, fewer fish were competent to settle at 10 dph (Table 4, Fig. 4B). In the most extreme pCO_2_ treatment only, fish exhibited diminished somatic growth relative to those at lower concentrations (Fig. 4C).

## Discussion

Otoliths exhibited diverse responses to treatment according to type and side. In response to increasing seawater pCO_2_, all three otolith types exhibited increasing perimeter and percent visible crystals, sagittae and lapilli exhibited increasing area and lateral development, and asterisci exhibited differences in shape. While the sagittae changed according to the same metrics regardless of side, the lapilli and asterisci changed according to different metrics depending on side. These differences reveal important things about the nature of the metrics under investigation; for example, while both sagittae responded to treatment by growing larger with more pronounced lateral faces, these effects were segregated according to side in the lapilli; this suggests that otolith area and lateral development are uncoupled rather than being two immutably conjoined metrics of growth. As such, it is often informative to investigate each otolith independently rather than investigating one type or pooling by type without regard to side. Among the 24 studies reviewed here that analyzed ocean acidification impacts on otolith morphology, five investigated lapilli (Table 1), none investigated asterisci, and eight segregated otoliths by side during morphometric analysis (at least six of which pooled them after observing no evidence of asymmetry) (Franke & Clemmesen, 2011; Munday et al., 2011a; Munday et al., 2011b; Maneja et al., 2013; Bignami, Sponaugle & Cowen, 2014; Mu et al., 2015; Perry et al., 2015; Réveillac et al., 2015; Martins, 2017; Jarrold & Munday, 2018). However, the results suggest that responses of one or two otoliths cannot necessarily be extrapolated to the rest of the otolith system.

Some regression models were disproportionately weighted by the 3,000 µatm pCO_2_/pH 7.30 treatment, as evidenced by the *p*-value change between the full model and truncated model with data from that treatment excluded. Although the *p*-values for all otolith morphological models increased with truncation (Table 4, attributable in part to a loss of power from fewer degrees of freedom), the p-values for the right lapilli and left asterisci models rose >0.05, indicating there is little to no evidence of pCO_2_ impacts on those otoliths except in conditions of aragonite undersaturation. For the lapilli, this exercise indicates an asymmetry of resilience: left lapilli are impacted by lower pCO_2_ concentrations, whereas right lapilli are not. Nevertheless, this still results in morphological asymmetry of lapilli at lower concentrations. For the asterisci, it indicates mutual resilience at lower concentrations, and morphological asymmetry at the highest concentration. Scenarios in which these relationships are ecologically relevant could include isolated coastal systems with poor mixing under additional pressure from eutrophication (Wallace et al., 2014; Fabricius, 2005) or volcanism (Vogel et al., 2015). However, lower concentrations are here considered more typical of the future ocean.

Researchers previously examined otolith development in teleost larvae reared under acidified conditions, and despite differences in methodology and model species, it is possible to draw comparisons. Notably, Munday et al. (2011b)’s study species (*Amphiprion percula*) enables intragenus comparison with *A. clarkii*. Here, the results are consistent with those of Munday et al. (2011b) and several others (Table 1) in that sagittae grew at elevated seawater pCO_2_. However, Munday et al. (2011b) observed growth in left sagittae only, whereas *A. clarkii* exhibited growth in both sagittae. The results are further consistent with six of those studies (Table 1) in that lapilli also grew at elevated pCO_2_ (albeit in the left ear only). Regarding otolith shape: the results are consistent with five studies (Table 1) in that otolith shape changed at elevated pCO_2_, albeit in left asterisci only, and only in conditions of aragonite undersaturation. A caveat: whereas most other studies reared fish from two or more genotypes (e.g., Munday et al., 2011a; Munday et al., 2011b; Bignami, Sponaugle & Cowen, 2013; Bignami et al., 2013; Bignami, Sponaugle & Cowen, 2014), the present study used one clutch of eggs produced by one broodstock pair. While this eliminated lineage as a potentially confounding variable in the analysis, care should be taken when comparing the results of this study with others or extrapolating to larger populations.

Some of the observed effects of seawater pCO_2_ on otolith growth in *A. clarkii*, including increasing area, perimeter, and lateral development, may be consequences of acid-base regulation triggered by respiratory acidosis. Fishes normalize internal pH disturbances by metabolic adjustment: blood plasma HCO${}_{3}^{-}$ is absorbed/retained and H^+^ excreted by modulating rates of transport across the gill epithelium. However, extracellular pCO_2_ and HCO${}_{3}^{-}$ remain elevated following pH adjustment, and excess HCO${}_{3}^{-}$ is imported to the endolymph where it becomes substrate for CO${}_{3}^{2-}$ aggregation, enhancing net otolith calcification (Checkley et al., 2009; Munday et al., 2011b; Heuer & Grosell, 2014).

Since otoliths are critical components of the ears and vestibular organs (Fekete, 2003; Moyle & Cech, 2004), ocean acidification-driven changes to otolith development may challenge sensory perception in *A. clarkii* and other teleosts (e.g., Munday et al., 2011b; Bignami et al., 2013; Bignami, Sponaugle & Cowen, 2014). Indeed, there is some empirical evidence outside the context of ocean acidification that marine fish exhibiting abnormal otolith morphology or asymmetry suffer diminished sensory ability. In terms of hearing, fish with larger, vateritic, and/or otherwise asymmetrical sagittae exhibited reduced sensitivity or deafness (Oxman et al., 2007; Gagliano et al., 2008; Browning et al., 2012); some presumably because vaterite is less dense than aragonite, thereby reducing otolith displacement amplitude and effectiveness in the inner ear (Bignami et al., 2013; Reimer et al., 2016). Although there remains no evidence of ocean acidification-induced vaterite replacement in otoliths, including in *A. clarkii*, there is some evidence for calcite replacement in sagittae and lapilli at elevated pCO_2_ (Coll-Lladó et al., 2018); calcite is similarly less dense than aragonite (Nakamura Filho et al., 2014). In terms of kinesthesia, some fish with abnormal and/or asymmetric sagittae/lapilli exhibited kinetoses (Söllner, 2003; Anken, Knie & Hilbig, 2017); however, other studies observed pCO_2_ impacts on otolith morphology without observing impacts on behavior (Bignami, Sponaugle & Cowen, 2013; Bignami, Sponaugle & Cowen, 2014; Shen et al., 2016). It is possible the pCO_2_ impacts on otolith morphology and asymmetry observed here could impair *A. clarkii* hearing and kinesthesia, but no sensory or behavioral assays were conducted, so hypotheses remain speculative.

In addition to corroborating reports of otolith growth along the *x* and *y* axes (i.e., increasing area and perimeter) in young teleosts in response to increasing seawater pCO_2_, there was evidence for pCO_2_-induced otolith growth along the *z*-axis (i.e., upward growth from the lateral face) in *A. clarkii*. Lateral development appears most conspicuous in sagittae, and linked to treatment in sagittae and right lapilli, although it was observable in asterisci as well. While lateral development occurs on the lateral face, which does not directly interact with maculae, it is possible this CaCO_3_ aggregation will increase otolith mass at a magnitude greater than that which is evident from increased 2-dimensional area and perimeter. Thus, sagittae exhibiting advanced lateral development may have a wider displacement amplitude independent of area and perimeter, enhancing auditory sensitivity (Bignami et al., 2013); however, displacement amplitude was not measured here. This hypothesis is independent of otolith composition, for which there was no evidence of having changed, but which undermined auditory sensitivity in some studies (Oxman et al., 2007; Browning et al., 2012; Reimer et al., 2016). Also, since lateral development appears to occur on only one face of the otolith (though the medial face was not investigated here, all otoliths were imaged convex-side up, which was invariably the lateral face), its center of mass likely changed as well, with unknown consequences for otic mechanics.

Some of the otoliths appear visibly smooth on the surface, while others appear rougher due to the exposure of aragonite table edges and similar crystal activity. Estimating percent visible crystals is akin to estimating otolith surface roughness. The observation that percent visible crystals increased with increasing pCO_2_ in sagittae, right lapilli, and left asterisci is consistent with the characterization of rough-type otoliths as abnormal in other species (Béarez et al., 2005; Ma et al., 2008; Browning et al., 2012). Increasing roughness could be a symptom of haphazard CaCO_3_ aggregation, evidence of altered protein matrix deposition, and/or a snapshot of an evolving CaCO_3_ crystal habit/polymorph baseline. While increasing roughness seems unlikely to affect otolith displacement amplitude, it could conceivably impact otolith-maculae mechanics with unknown consequences for function; in some fish, otoliths are observed to be rough on the ventral end only, driving maculae deformation by hooking them to the otolith surface (Ohnishi et al., 2002). More research concurrently investigating fish behavior, ocean acidification-induced otolith roughness, and maculae displacement is needed to explore this hypothesis.

As might be expected when rearing many hundreds of fish in the most delicate early stages of development, *Amphiprion clarkii* larvae experienced substantial mortality throughout the experimental trial. Although there is evidence in the literature of acute CO_2_ toxicity in larval teleosts, this is typically observed at pCO_2_ levels far exceeding those evaluated here (i.e., >48,000 uatm) (Kikkawa, Ishimatsu & Kita, 2003; Ishimatsu et al., 2004; Kikkawa, Kita & Ishimatsu, 2004); this is consistent with the absence of evidence that treatment instigated fish mortality. The sublethal impact of pCO_2_ on settlement competency at 10 dph, however, was dramatic. There is some evidence of inhibited larval growth following delays in metamorphosis (Victor, 1986; McCormick, 1999), and some degree of inhibited growth was observed here, but the present analysis should be interpreted with caution due to the following caveats: (i) 10 dph was selected as the onset of metamorphosis and presumed settlement competency, but this threshold may be arbitrary to metamorphosis and settlement completion; (ii) metamorphosis and response to settlement cues may decouple at elevated seawater pCO_2_ (Rossi et al., 2015), complicating metamorphosis as a reliable indicator for settlement competency; (iii) indeed, laboratory-reared fish were unexposed to settlement cues including reef sounds, lunar phase, and anemone presence; (iv) otolith microstructure evidence of settlement competency was not investigated; (v) inhibited larval growth due to delayed metamorphosis isn’t known to translate to inhibited growth post-settlement (McCormick, 1999). Finally, the impact on somatic growth was marginal: the reduction in fish standard lengths with increasing pCO_2_ amounts to a small fraction of fish standard lengths, and this effect was only relevant in conditions of aragonite undersaturation.

## Conclusions

This work corroborates evidence of otolith growth and altered shape with increasing seawater pCO_2_ reported for other taxa in a novel taxon, *Amphiprion clarkii*. In addition, it reports evidence of increasing otolith lateral development and surface roughness with increasing pCO_2_. Impacts were observed in all otolith types, including the previously uninvestigated asterisci. Each otolith type and side were investigated independently, indicating asymmetrical responses of lapilli and asterisci to pCO_2_. The experimental design and analysis facilitated construction of pCO_2_ dose–response curves, which were created for all otolith types and sides in *A. clarkii* excepting right asterisci. These curves outline changes to multiple morphometric and mineralogical variables and may be leveraged to predict responses to pCO_2_ conditions not investigated here. These responses could impact auditory and/or vestibular sensitivity in teleosts, adding to previous observations and hypotheses involving sagittae and lapilli. In summary, the work adds to the existing knowledge base regarding otolith response to ocean acidification, which may aid in predicting and preserving teleost fitness in the near-future ocean.

##  Supplemental Information

10.7717/peerj.6152/supp-1 Data S1 Tank means for statistical analysis Tank Means for Statistical Analysis. Includes SL (mm); Area/SL, Perimeter/SL (um/mm); circularity (dimensionless); lateral development (scale of 1–5); percent visible crystals (scale of 5–50%); as well as tank counts of stocking density, settled, unsettled, dead (mort), and surviving (remaining) fish at the end of the experimental trial. This file is in database format and is intended to be subset by otolith type/side (“OTIE”).

10.7717/peerj.6152/supp-2 Data S2 Raw fish-level data Raw Data (Fish-Level). Includes SL (mm); Area/SL, Perimeter/SL (um/mm); circularity (dimensionless); lateral development (scale of 1–5); percent visible crystals (scale of 5–50%).

10.7717/peerj.6152/supp-3 Supplemental Information 1 Statistical analyses Annotated R code used to run statistical analyses on fish and otolith morphometric data.

10.7717/peerj.6152/supp-4 Supplemental Information 2 Micrograph scoring rubric Rubric used for training and guiding scanning electron micrograph readers through scoring of four otolith mineralogical metrics. NOTE: “Core development” in the rubric has been renamed “lateral development” in the manuscript. In the rubric, “core” refers not to the otolith’s core but to the center of its lateral face.

## Additional Information and Declarations

### Data Availability

The following information was supplied regarding data availability: Raw data are provided in the Supplemental Material; BCO-DMO (OA-OTO MIN; https://www.bco-dmo.org/project/2240); and GitHub (https://github.com/edfreeburg/RSS-feed-DigitalAquaticsRKE/blob/master/DA_RSS_Multiple.pl; https://github.com/edfreeburg/RSS-feed-DigitalAquaticsRKE/blob/master/rss_data.m; https://github.com/edfreeburg/MATLAB-Image-Analysis/blob/master/Morpho.m).
