# Supplementary material for: Ocean acidification alters morphology of all otolith types in Clark’s anemonefish (Amphiprion clarkii)
Source: PeerJ. 2019 Jan 7;7:e6152. doi: 10.7717/peerj.6152 (PMC6327886; doi:10.7717/peerj.6152)

<u>CHARACTERISTIC #1 - CRYSTAL HABIT</u>

Crystal habit has to do with shape of a crystal. You should focus on the whole otolith though most of the crystal structure will be in the “core” of the otolith. Below are the habits and pictures of the “ideal” shape.

Orthorhombic

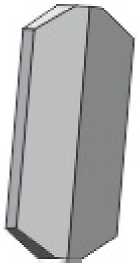

Hexagonal

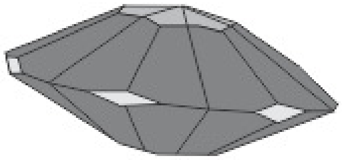

Acicular

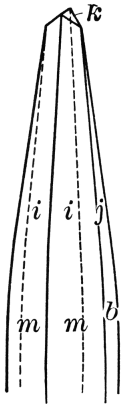

acrystalline
No obvious crystals

Amorphous
Small “blubs” of material

<u>To Score the images, identify the DOMINANT habit:</u>

1 = orthorhombic
2 = acicular
3 = hexagonal
4 = acrystalline (no crystals)
5 = amorphous

CHARACTERISTIC #2 – MINEROLOGY

Here we will use crystal habit to identify relative percentages of minerals using the following key:

Aragonite Vaterite

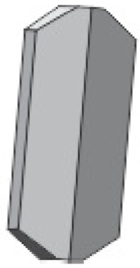

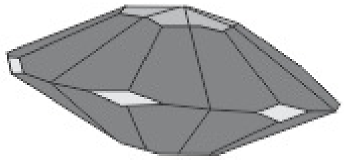

Score images as follows:

1 = completely aragonite
2= vaterite ≤ 33%
3 = vaterite 33% to 66%
4 = ≥ 66% vaterite.
5 = no visible crystals

CHARACTERISTIC #3 - % VISIBLE MINERALS
Using your best estimate, what is the relative % of visible crystals? Enter into Box.

ESTIMATING PERCENTAGES
Below are images with the relative % of dark material noted. When you look at these figures examine the balance of white space to dark space use the balance to estimate % dark.

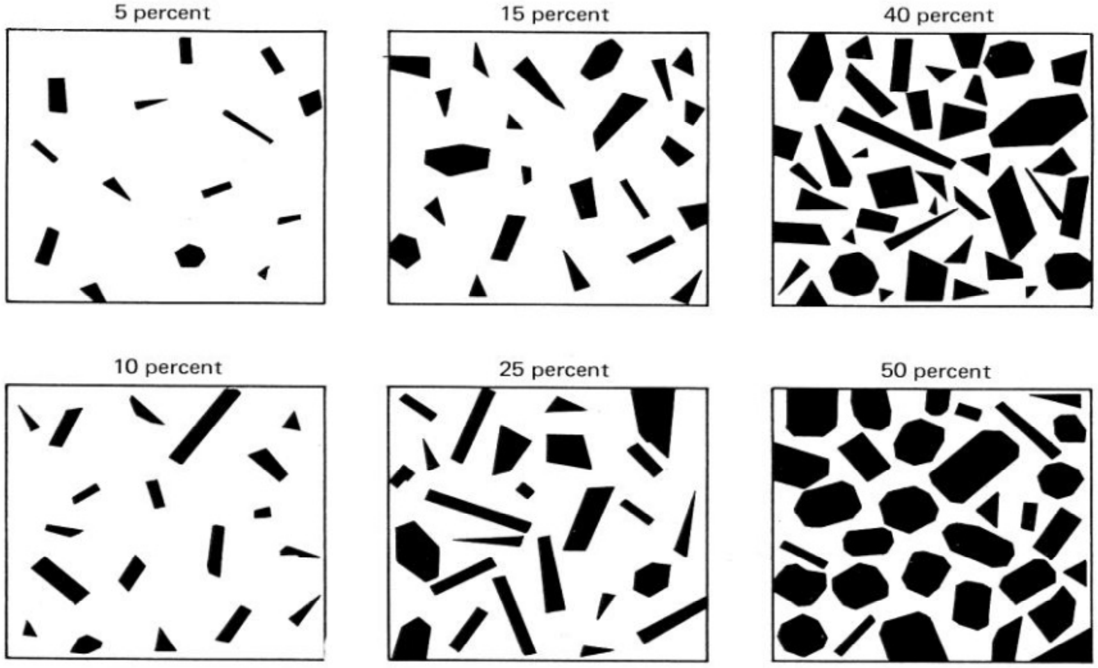

Using the above figure as an example you will be asked to evaluate the % of a particular characteristic in images of fish ear stones (otoliths).

CHARACTERISTIC #4 - CORE DEVELOPMENT

Rank core crystal development using the scheme shown below (1-5)

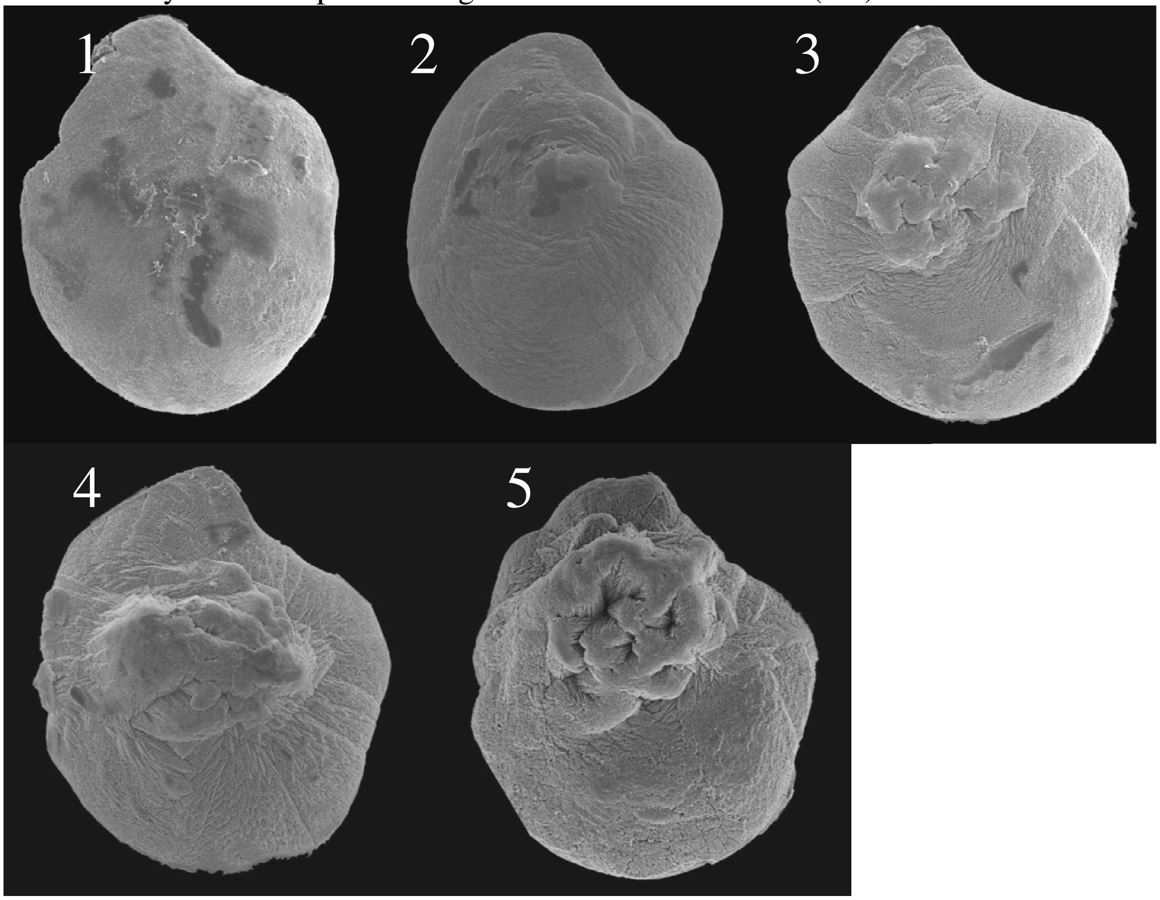

Supplement: Supplemental Information 2 — Rubric used for training and guiding scanning electron micrograph readers through scoring of four otolith mineralogical metrics. NOTE: “Core development” in the rubric has been renamed “lateral development” in the manuscript. In the rubric, “core” refers not to the otolith’s core but to the center of its lateral face. [file peerj-07-6152-s004.pdf]
